# Role of Social Determinants of Health in COVID-19 Recovery: A Qualitative Study

**DOI:** 10.1001/jamanetworkopen.2024.53261

**Published:** 2025-01-06

**Authors:** Neelima Navuluri, Nrupen A. Bhavsar, Vivian Chen, Margaret Falkovic, Laura J. Fish, Lauren Gray, Christina Makarushka, Laura Mkumba, Hnin Thuzar Lwin, Auston Stiefer, Deepshikha Charan Ashana

**Affiliations:** 1 Department of Medicine, Duke University, Durham, North Carolina; 2 Duke Global Health Institute, Duke University, Durham, North Carolina; 3 Department of Surgery, Duke University, Durham, North Carolina; 4 Department of Biostatistics & Bioinformatics, Duke University, Durham, North Carolina; 5 Duke University School of Medicine, Durham, North Carolina; 6 Duke Cancer Institute, Duke University, Durham, North Carolina; 7 Department of Family Medicine & Community Health, Duke University, Durham, North Carolina; 8 Department of Population Health Sciences, Duke University, Durham, North Carolina; 9 Duke-Margolis Center for Health Policy, Duke University, Durham, North Carolina

## Abstract

**Importance:**

Health systems are increasingly required to conduct health-related social needs screening. However, how social resources negatively and positively affect recovery from acute illnesses, such as COVID-19, is incompletely understood.

**Objective:**

To examine how social determinants of health (SDOH) influence recovery from COVID-19.

**Design, Setting, and Participants:**

In this qualitative study, patients were recruited for semistructured interviews from a post–COVID-19 pulmonary clinic at a southeastern US academic medical center between November 2022 and March 2023. Caregivers were included in dyadic interviews when available. Interviews queried participants about SDOH domains, as defined by the US Centers for Disease Control and Prevention, and their relationship with COVID-19 recovery.

**Main Outcome and Measures:**

Phenomenologic analysis identified themes characterizing participant perspectives on the influence of SDOH on COVID-19 recovery.

**Results:**

A total of 24 interviews were conducted: 10 (42%) with patient-caregiver dyads, 13 (54%) with patients alone, and 1 (4%) with 2 patients who also served as each other’s caregiver. Most participants were female (18 patients [72%] and 6 caregivers [60%]). The median age of patients was 57 years (IQR, 44-61 years) and of caregivers was 47 years (IQR, 39-62 years). Three cross-cutting themes that overlapped SDOH domains were identified. Participants noted that innovative mobilization of social resources (eg, policies to secure income during time away from work) supported recovery from COVID-19 illness, but destabilization and change introduced by illness (eg, disrupted social support networks) and mistrust of previously established institutions (eg, public health misinformation) hindered recovery.

**Conclusions and Relevance:**

Participants identified 3 distinct SDOH domains positively and negatively influencing recovery from COVID-19 illness. The findings suggest that longitudinal, multidomain data on SDOH are needed to best address barriers and identify resources for patients recovering from acute illness and may help determine opportunities for system- and policy-level interventions that can mitigate the influence of long-standing structural inequities on health.

## Key Points

**Question:**

How do social determinants of health (SDOH) impact recovery from COVID-19?

**Findings:**

In this qualitative study including 24 interviews with patients and patient-caregiver dyads, participants noted that innovative mobilization of social resources (eg, policies to secure income during time away from work) supported recovery from COVID-19 illness. In contrast, destabilization and change introduced by illness (eg, disrupted social support networks) and mistrust of previously established institutions (eg, public health misinformation) hindered recovery.

**Meaning:**

The findings suggest that SDOH both support and hinder recovery from COVID-19 and may inform how health systems integrate social and medical care.

## Teaser

This qualitative study explores how patients with COVID-19 and their caregivers perceive the role of social determinants of health in their recovery from COVID-19.

## Introduction

Syndemic theory explains how distinct biological and social epidemics interact to worsen health outcomes. For example, rates and severity of COVID-19 illness were impacted by dynamic social conditions, including access to health care, population and housing density, changes in labor markets, and family and community support, which interacted to produce worse health outcomes among structurally marginalized populations.^1,2,3,4,5^ These interactions impacted not only the experience of acute COVID-19 infection but also the recovery period following acute illness, a time often marked by new functional, cognitive, emotional, social, and financial challenges.^6,7,8,9^

A deeper understanding of the mechanisms through which socioenvironmental conditions exacerbate illness to form a syndemic not only is important in its own right but also is increasingly critical to informing care and policy.^2,10^ The Centers for Medicare & Medicaid Services mandated that hospitals report the number of inpatients who are screened for social determinants of health (SDOH) beginning in 2024.^11^ Yet, how hospitals and policymakers will use these data to support population health both during and after acute illness is not known. Data on how SDOH affected recovery from COVID-19, especially among those with persistent health sequelae, can be useful in informing future care and policy.^12,13,14,15,16,17^

We used COVID-19 as a model to understand how SDOH may support or hinder recovery from acute illness. We used qualitative methods, including interviewing patients who had experienced COVID-19 and their caregivers, because these allow an in-depth exploration of SDOH and the ways in which they may interconnect in participants’ own words.^18^

## Methods

### Parent Study

In this qualitative study, we conducted interviews from a subsample of patients who presented to a post–COVID-19 pulmonary clinic at Duke Health and enrolled in a prospective cohort study (n = 151) to follow clinical and patient-reported outcomes.^19,20,21^ The parent study enrolled participants between June 2020 and March 2022 who had a positive SARS-CoV-2 antigen or polymerase chain reaction test result. Individuals who took part in blood conservation programs that would preclude providing study samples were excluded. Patients were generally referred to the clinic by a health care professional but could also be self-referred regardless of insurance status. Further details about this cohort and clinical outcomes are published elsewhere.^19,20,21^ This study received approval from the Duke University Health System institutional review board, and all participants provided written informed consent, including to publish quotes that were deidentified. This study adhered to the Standards for Reporting Qualitative Research (SRQR) reporting guideline.^22^

### Study Setting, Design, and Participants

This study took place in Durham, North Carolina, and recruited participants from Duke Health, a large academic medical center in the southeastern US. The Duke Health system sees nearly 5 million outpatients a year, with the majority of patients coming from Durham, Wake, and Orange counties in North Carolina. These counties are demographically and socioeconomically diverse, with approximately 22% identifying as Black or African American and 11% of individuals experiencing poverty.^23^ North Carolina was ranked 21st for highest death rates from COVID-19 in the US in 2022.^24^

We purposively sampled participants for semistructured interviews based on self-reported race (categories included Black or African American, White, and other [not further specified to preserve participant anonymity]), ethnicity (Hispanic or Latino, not Hispanic or Latino), neighborhood socioeconomic status as measured by the Area Deprivation Index (ADI) of the participant’s home address, and dyspnea as measured by the Borg Dyspnea Scale.^25,26,27^ Our goal was to obtain diverse perspectives from an equal proportion of participants who identified as Black or African American or as White (numbers of participants in other racial groups were small), lived in neighborhoods spanning a broad range of socioeconomic conditions, and had varying levels of dyspnea or post–COVID-19 respiratory symptoms. Given the range of ADI and Borg scores in the original cohort, we focused initial recruitment efforts on those with the highest ADI and Borg scores. To enhance recall of COVID-19 recovery experiences, patients were asked to identify the caregiver who was most instrumental in their COVID-19 recovery for participation in dyadic interviews. We compensated each participant with $50.

### Data Collection

The interview guide was informed by a framework of SDOH domains defined by the US Centers for Disease Control and Prevention.^15^ These include economic stability, health care access and quality, social and community context, education access and quality, and neighborhood and built environment. Questions focused on the impact of these domains on COVID-19 recovery (eAppendix 1 in Supplement 1). The interview guide was pretested with 4 individuals and refined prior to participant recruitment. Patients and caregivers (if a caregiver was available) were interviewed together via either videoconferencing software or telephone by experienced female qualitative interviewers (M.F., L.J.F., and L.M.) between November 2022 and March 2023. If a caregiver was not identified or available, patients were interviewed individually. Caregivers were asked to complete a brief demographic survey including information on age, medical history, insurance, occupation,^28^ and COVID-19 history. All interviews were audio-recorded and transcribed verbatim. The sample size was based on the principle-of-information power—stopping the recruitment of new participants when sufficient information relevant for the study was achieved based on research aims, specificity of the sample, interview quality, and the SDOH framework.^29^

### Data Analysis

A codebook was iteratively developed based on the SDOH framework and preliminary coding of transcripts using NVivo, version 12 (QSR International). One-third of the transcripts (n = 8) were independently coded by at least 2 of 3 coders (V.C., H.T.L., and A.S.) to further refine the codebook.^30,31^ Differences in coding were resolved by group discussion until consensus was reached. A final codebook was developed (eAppendix 2 in Supplement 1), and the remaining transcripts (n = 16) were coded by a single investigator (N.N. or D.C.A., primarily). Questions or concerns that arose during coding were discussed at team meetings. Data were analyzed with NVivo, version 12, using both deductive and inductive analyses. Deductive content analyses identified facilitators and barriers to recovery within each SDOH domain, and inductive thematic analysis identified cross-cutting, emerging themes using a phenomenological approach.^32^ In addition, in an exploratory analysis to assess whether the influence of SDOH varied by neighborhood disadvantage, codes in each SDOH domain were cross-tabulated by valence (positive, negative, or ambivalent, as assigned independently by 2 investigators [V.C., D.C.A.]) and participants’ ADI compared with the median ADI (median, 3; lower disadvantage, 1-3; higher disadvantage, 4-10).

## Results

### Participants

We conducted 24 interviews: 10 (42%) with patient-caregiver dyads, 13 (54%) with patients alone, and 1 (4%) with 2 patients who also served as each other’s caregiver (categorized hereafter as *patients*). Interviews were conducted using videoconferencing software (15 [62%]) or by telephone (9 [38%]). Demographic characteristics of the 25 patient and 10 caregiver participants are shown in Table 1. The median age of patients was 57 years (IQR, 44-61 years) and of caregivers was 47 years (IQR, 39-62 years). Eighteen patients (72%) were female, and 7 (28%) were male; 6 caregivers (60%) were female, and 4 (40%) were male. Most participants had completed college or university (patients: 19 [76%]; caregivers: 7 [70%]) and had health insurance (patients: 24 [96%]; caregivers: 9 [90%]). Among patients, 14 (56%) identified as Black or African American, 10 (40%) as White, and 1 (4%) as other race; 1 (4%) identified as Hispanic or Latino and 24 (96%) as not Hispanic or Latino. Among caregivers, 4 (40%) identified as Black or African American, 6 (60%) as White, and none as other race; none identified as Hispanic or Latino and 10 (100%) as not Hispanic or Latino. The relationship of caregivers to patient participants included spouse (5 [50%]); parent (2 [20%]); sibling, aunt, or uncle (2 [20%]); and formal health care giver (1 [10%]).

**Table 1.** Characteristics of Patients and Their Caregivers

Characteristic	Participants^a^	
	Patients (n = 25)	Caregivers (n = 10)
Age, median (IQR), y	57 (44-61)	47 (39-62)
Sex at birth		
Female	18 (72)	6 (60)
Male	7 (28)	4 (40)
Race		
Black or African American	14 (56)	4 (40)
White	10 (40)	6 (60)
Other^b^	1 (4)	0
Ethnicity		
Hispanic or Latino	1 (4)	0
Not Hispanic or Latino	24 (96)	10 (100)
Relationship to participant		
Spouse	NA	5 (50)
Formal health care giver	NA	1 (10)
Parent	NA	2 (20)
Other (sibling, aunt, uncle)	NA	2 (20)
Highest level of education		
Secondary school (9th-12th grade)	6 (24)	3 (30)
College or university	14 (56)	5 (50)
Graduate school	5 (20)	2 (20)
Insurance type		
Commercial	19 (76)	7 (70)
Medicare and/or Medicaid	5 (20)	2 (20)
Uninsured	1 (4)	1 (10)
Occupation^c^		
Computer, engineering, science	2 (8)	0
Health care	7 (28)	3 (30)
Health care support	0	1 (10)
Legal, social, education	4 (16)	1 (10)
Management or business	3 (12)	0
Not working	4 (16)	2 (20)
Sales and office	2 (8)	2 (20)
Transport, maintenance	3 (12)	1 (10)
Vapor, gas, dust, or fume exposure^d^		
Yes	4 (16)	NA
No	21 (84)	NA
Chronic medical conditions		
Hypertension	14 (56)	1 (10)
Diabetes	8 (32)	4 (40)
Mental health condition (eg, anxiety)	NA	3 (30)
Thyroid disease	3 (12)	1 (10)
Other^e^	25 (100)	1 (10)
None	0	3 (30)
COVID-19 diagnosis in the past 3 y		
Any	25 (100)	7 (70)
Symptomatic, not hospitalized	4 (16)	6 (85)
Hospitalized on general ward	19 (76)	0
Hospitalized in ICU	2 (8)	0
Asymptomatic	0	1 (14)
Borg Dyspnea Scale at 6 wk after COVID-19 diagnosis, median (IQR)^f^	1 (0-3)	NA
ADI decile		
1 (Lowest level of disadvantage)	3 (12)	NA
2	8 (32)	NA
3	4 (16)	NA
4	3 (12)	NA
5	2 (8)	NA
6	0	NA
7	0	NA
8	2 (8)	NA
9	1 (4)	NA
10	2 (8)	NA
PASC symptoms, No.		
1	17 (68)	NA
2	6 (24)	NA
3	2 (8)	NA
Symptoms reported 6 wk after COVID-19 diagnosis		
Pulmonary	21 (84)	NA
Cardiovascular	6 (24)	NA
ENT	0	NA
Neurologic	5 (20)	NA
Psychologic	3 (12)	NA

Abbreviations: ADI, Area Deprivation Index; ENT, ear, nose, and throat; ICU, intensive care unit; NA, not applicable; PASC, postacute sequelae of COVID-19.

^a^ Data are presented as number (percentage) of participants unless otherwise indicated.

^b^ Other race is not further specified to preserve participant anonymity.

^c^ Based on US Bureau of Labor Statistics Occupational and Employment Groups.^28^

^d^ Participants were asked whether they had vapors, gas, dust, or fume exposure, which includes chlorine; ammonia; fire; refineries; diesel exhaust; grain, cotton, or wood dust; animal feeds; or fumes from batteries, welding, metals, or fiberglass.

^e^ Includes anemia, asthma, breast cancer, chronic kidney disease, chronic obstructive pulmonary disease, gastroesophageal reflux, inflammatory bowel disease, interstitial lung disease, leukemia, lupus, lymphoma, obstructive sleep apnea, and supraventricular tachycardia.

^f^ On 10-point scale, with 0 indicating none and 10, maximal. Two patients answered NA.

The median ADI among patients was 3 (IQR, 2-4); 15 patients (60%) were from lower-disadvantage neighborhoods (ADI of 1-3) and 10 (40%) from higher-disadvantage neighborhoods (ADI of 4-10). With regard to their initial COVID-19 illness, 21 patients (84%) were hospitalized, and all 25 patients had at least 1 postacute sequela of a SARS-CoV-2 symptom. These included pulmonary (21 [84%]), cardiovascular (6 [24%]), neurologic (5 [20%]), and psychological (3 [12%]) symptoms.

### Participant-Identified Facilitators and Barriers to Recovery

Participants highlighted several facilitators and barriers to recovery within each SDOH domain (Table 2). For example, having accommodations for disabilities at home or in public spaces (neighborhood and built environment domain), connections with church groups and community support (social and community context domain), paid time off or flexible work arrangements (economic stability domain), and grocery delivery or food drop-offs by members of their social network (food access and quality domain) were all helpful during their recovery.

**Table 2.** Social Resources or Needs Discussed by Interview Participants

SDOH domain	Resources	Challenges
Neighborhood and built environment	Safe, walkable neighborhood Living in a home with accommodations or having alternate places to live (eg, family member’s home without stairs) Living in a home with ability to quarantine from others and access to separate restrooms Accommodations in public spaces required by Americans With Disabilities Act	Distance from parking lot to workplace Neighborhood safety concerns
Social and community context	Having a caregiver Prayers from faith community; having religious faith Ability to stay in contact virtually with family members and friends	Being a caregiver while dealing with own illness Disruption of social network due to deaths of family or friends Being ostracized due to COVID-19 illness Social anxiety due to fear of contracting COVID-19
Economic stability	PTO, including pooling and redistribution of PTO among coworkers; supportive federal programs such as FMLA and PPP Social Security benefits among older adults Remote or part-time work Enforcement of masking requirements in workplace	Loss of income due to contract work, termination, new disability, or death of income-earning family member Unforeseen health-related expenses High-infectious-risk workplace
Health care	Home-based medical care, such as having an oxygen concentrator and visiting nurses or physical therapists Health department telephone-based wellness checks Trusted clinicians Telehealth appointments Medication drop-off by friends and family	Limited supply of investigational drugs Limited number of outpatient facilities seeing patients with COVID-19 Extended wait times for specialist care Overcrowded emergency departments Health care personnel staffing shortages Dismissive clinicians
Food access and quality	Grocery delivery or pick-up services Meals shared by members of social network Food drop-off by members of social network	Lack of stamina to obtain or carry groceries Difficulty maintaining nutrition due to loss of taste Distance from grocery stores in rural areas
Education access and quality	Being employed in health care Staying up to date with new COVID-19 vaccines via news sources and social network	Conflicting, evolving, or incomplete public health information

Abbreviations: FMLA, Family and Medical Leave Act; PPP, Paycheck Protection Program; PTO, paid time off; SDOH, social determinants of health.

In contrast, unsafe neighborhoods (neighborhood and built environment domain), disruption of social networks due to deaths of loved ones (social and community context domain), unforeseen health-related expenses (economic stability domain), dismissive clinicians or extended wait times for specialist care (health care domain), and conflicting or incomplete public health information (education access and quality domain) hindered recovery. No notable quantitative or qualitative differences were noted after stratification by participant ADI (eTable in Supplement 1).

### Themes Spanning SDOH Domains

Three cross-cutting themes were identified that overlapped SDOH domains: innovative mobilization of resources, navigating the destabilization and change introduced by illness, and mistrust of previously established institutions (Table 3). Each theme describes how SDOH collectively influenced recovery from COVID-19 and other acute illnesses.

**Table 3.** Emergent Themes and Additional Exemplary Quotes

Theme	Exemplary quotes (participant details)
**Mobilization and innovation of resources**	
Social resources	“I really did not go like venture out, go shopping, go to the grocery store. They brought the stuff for me or they cooked a lot of stuff for me too. A lot of my friends, my coworkers, my [name of institution] friends you know, when that doorbell ring, but they’ll be, they’ll ring the doorbell and run to the car, they brought me a plate, they brought me groceries you know, they brought me water you know.” (Interview 14, P, ADI of 4)
	“Shout out to our church out there. They were amazing.... They were able to help out a lot more there for us if we needed anything. Just ask, right? They were probably one of the biggest helps, and then some of our good neighbors and resources.” (Interview 21, P and C, ADI of 1)
	Patient: “I think it was through the Salvation Army actually—we were giving out things like Tylenol and Motrin.” Caregiver: “Oh yeah the churches, you remember they would give all the Tylenol, they would give out cold and flu medicine.” (Interview 4, P and C, ADI of 10)
Financial resources	“I was able to access my medical leave, so I could be out of work without…any repercussions from it. They were doing the 80 hours of leave. I was out more than 80 hours, but at least…those 2 weeks were paid...It was just something they did for all employees because of the pandemic, and I think because so many people were out of work because of it. It was something that they put in place at the time.” (Interview 2, P and C, ADI of 3)
	“I spent that first 2 months of the year part time on FMLA because of I guess you know, the policies for dealing with COVID patients and what is it…[name of institution] wasn’t paying like my salary, it was also being refunded from COVID patients. So I got caught in the middle of all of that stuff. It was not like they were paying my salary. So much of it came from that COVID policy. CDC policy.” (Interview 14, P, ADI of 4)
**Navigating the destabilization and change introduced by illness**	
Fear of human connection	“I kind of purposely started isolating myself because I did not like to go out. Because I got so sick, and it was very scary, and I couldn’t figure out how I ended up catching COVID.” (Interview 3, P and C, ADI of 2)
	“You won’t believe how careful we were not to let anybody breathe anywhere near us. It was so frightening because a lot of people that had COVID died and we knew that you better use your brain power and not get yourself exposed to anything. You won’t believe we didn’t even go in church. We drove up to the parking lot and sat in the car. And in the grocery store, you went in and out and you did not put yourself in a position to be around anybody that could give you COVID.” (Interview 8, P and C, ADI of 8)
Disruption of social network	“I came back home [from the hospital] and unfortunately [the next day] I had to travel to my parents. Because my dad was put on a medically induced coma. There was nothing else the doctors could do. After getting out of the hospital just have that stress of my dad being in the hospital on a ventilator and then later on him passing away and having to deal with the funeral stuff and then obviously afterwards just like grieving. Just thinking of what the future will hold for my family.” (Interview 24, P, ADI of 10)
	“It’s like I wish I had my life back where I could go exercise, where I could run where I can enjoy my friends. I don’t have that anymore. Things are different, even society is different and it’s not just me, I have lots of friends, I lost a lot of friends I worked with at [location] who died. I had a lot of family members who died so it was emotional.… My neighborhood is sad. My next-door neighbor she just turned 50. She died. Her husband came home and found her in the house dead and it was COVID related.” (Interview 14, P, ADI of 4)
Uncertain health state	“I’m trying to remember what ‘back to normal’ is at this point.” (Interview 3, P and C, ADI of 2)
	“Because I’m definitely not what I was prior to COVID. I’m just not. Even with me going back to work, even after the surgery, my pulmonologist is saying that there’s nothing else to do medically. There’s nothing else for me to do. I understand that, but at the same time, I don’t breathe like I used to. I can’t breathe like I used to. Like, you know, what happens?” (Interview 2, P and C, ADI of 3)
**Mistrust of established institutions**	
Unreliable protection and recommendations	“Number one is feeling you can trust your medical care team. Like I said, when I went to the first hospital, and they decided to send me home…. I was thinking, I just heard him say how advanced this is, but they’re still going to send me home, and can I really trust him to give me adequate medical care?” (Interview 3, P and C, ADI of 2)
	“But, of course the contradictions in, and the advice we were getting was kinda for me—it was like why don’t they just stay quiet until they know what’s going on? You know, rather than tell us 1 thing 1 day and another the next. You know, ‘masks don’t work.’ ‘Masks work.’ You know that kind of thing, and quite honestly, the medical staff, they knew nothing, you know…. When I first got sick…I thought I was gonna have a heart attack…so we called 911 and there was no answer.” (Interview 16, P and C, ADI of 2)
Dismissal by clinicians	“When I went to see the pulmonologist…even though I was having all these symptoms, still the shortness of breath and fatigue, he chalked it all up to me basically being ‘fluffy’ or overweight. That was really hard for me emotionally, because my weight had really not changed at that point and I knew what my baseline was...I knew something was not right…. And I know that now because they’ve resolved…. I just felt very unheard…. I always felt like some of the health care providers I saw postdischarge felt like I was trying to get something out of it, or to be able to stay out of work, and that certainly was not my end game. My end game was just to feel better.” (Interview 1, P and C, ADI of 5)
	“I was in that ED, there was time, I was the only patient for hours, and I was complaining to the nurse, and she’s like, ‘Sir, we’ll get to you when we get to you.’” (Interview 11, P and C, ADI of 3)
Dissolution of social contract	“The experience I had with COVID. I wish that on nobody. It’s killed a lot of people out there. But they ain’t taking their shots. Scared.” (Interview 13, P and C, ADI of 1)
	“We still had people that were not wearing masks. Now it is a small percentage of them. But the store did not say that you can’t come in. They still never stopped them from being in the stores with other people. I think that was the one thing that surprised me is that still not everybody was doing what they were supposed to do with the masks.” (Interview 6, P, ADI of 1)

Abbreviations: ADI, Area Deprivation Index; C, caregiver; CDC, Centers for Disease Control and Prevention; ED, emergency department; FMLA, Family and Medical Leave Act; P, patient.

#### Mobilization and Innovation of Resources

The first theme details mobilization and innovation of financial, health care, and social resources. Innovation was often described in terms of new federal and employer policies that contributed to maintenance and replenishment of financial resources. These included policies around sick and medical leave, paid time off, and medical expense coverage, which provided both financial and mental relief for participants.

“So, there was a bucket of money essentially for people that had gotten COVID so they could help cover them if they didn’t have PTO [paid time off]…. And that was available, I think company-wide and a limited funding, obviously they don’t have unlimited money, but they were able to help people out during that time.” (Interview 21, patient and caregiver, ADI of 1)

Another major innovation centered on telemedicine services, which became more widely available during the pandemic. Many participants noted how their access to care was positively impacted by being able to connect with physicians virtually.

“Yeah with my doctor’s appointments, like I did it through Zoom, which is really good.” (Interview 6, patient, ADI of 1)

“I had a virtual appointment with my surgeon in Florida, and he said this isn’t how you should be feeling.” (Interview 8, patient and caregiver, ADI of 8)

Innovation around food and grocery delivery also was seen as useful. While not novel, these programs and resources were heavily mobilized and used over an extended course of time.

“I still was limited by getting around. So, I utilized the delivery options from [grocery stores]. Then on days that I wasn’t feeling so well, and if I needed a few things, or if I was feeling fatigued, I would just use the deliveries.” (Interview 12, patient, ADI of 3)

Other resources that participants used included community programs, such as Meals on Wheels, churches, and friends who helped to provide food, safety, medicines, money, and moral support during the COVID-19 pandemic. These resources were also mobilized in new and unique ways. For example, 1 participant described how they adapted gathering with their friends when they had to socially distance.

“I had 2 really good friends who you know I could not visit…so I would drive over to [place] and sit in the parking deck and I had a friend who drove from [town] and one who would drive from [town] and they parked on either side of me…and one of them would go in and buy breakfast for all of us, and we’d sit there [outside in separate cars] and eat.” (Interview 3, patient and caregiver, ADI of 2)

#### Navigating the Destabilization and Change Introduced by Illness

The second theme encompasses 3 subthemes, including fear of human connection, disruption of social networks, and uncertain health state. Regarding fear of human connection, participants discussed how having had COVID-19 led them to avoid interacting with others. For example, 1 patient participant shared the following:

“[My family] weren’t coming around because they didn’t want COVID you know and I didn’t want them to catch COVID. My life just changed…. I trust nobody because COVID is not a good feeling…. I don’t like being around people now.” (Interview 5, patient, ADI of 5)

Others shared examples of being ostracized by others due to their COVID-19 illness.

“Being shunned from my neighbors…. It would be almost 12 months before my family members and my friends, anyone felt safe to come to the living room, but if they came, they would stay maybe 10 minutes and they’re near the door, ready to run if I came too close.” (Interview 14, patient, ADI of 4)

Social networks were further disrupted as a result of deaths of family and friends from COVID-19 and other illnesses. These deaths weakened a support structure that participants normally relied on during times of illness.

“Just seeing all the people in my neighborhood, elderly people who passed away. That was hard. [In the] first year, I had 8 family members and maybe 2 friends pass away from COVID. And the second year, I think 6 altogether…. Just the family itself not being able to really care for each other the way we normally would care if we were sick. Everybody was kind of caring for themselves…we couldn’t really commune together.” (Interview 18, patient, ADI of 8)

Participants expressed anxiety about their future health, not knowing if or to what extent their symptoms would improve. As 1 participant questioned,

“Am I going to have to live with this the rest of my life? How am I going to figure out how to manage my daily cares, so that I can work and be a wife and a mom?” (Interview 1, patient and caregiver, ADI of 5)

#### Mistrust of Established Institutions

The final theme encompasses subthemes of unreliable protection and recommendations, dismissal by clinicians, and dissolution of social contracts. Participants shared how mixed messaging around COVID-19 prevention recommendations, such as masking, led to them feeling they could not rely on public health communication. One participant also shared how they did not trust the quality of protective resources their employers provided.

“Well, they [employer] provide sanitizer, but I don’t trust it. It might be watered down. No, I don’t trust what they provide.” (Interview 5, patient, ADI of 5)

Participants and their caregivers also detailed how clinicians dismissed their symptoms and concerns. These negative experiences led to mistrust in their care teams and the health system more broadly.

“Like I said, when I went to the first hospital, and they decided to send me home, I was thinking, I just heard him say how advanced this is, but they’re still going to send me home, and can I really trust him to give me adequate medical care?” (Interview 3, patient and caregiver, ADI of 2)

These experiences extended beyond individual interactions to the community writ large, with several participants discussing how there was a dissolution of an implied social contract during COVID-19 when it came to protection measures like masking or vaccination.

“As a country we just think we’re above it, whatever that means…. So, it’s not gonna happen to us…. And then you do get the people, the individuals, ‘I’m not wearing a mask. I don’t care what you say.’ That is the selfishness…. So, when I hear people making ridiculous claims about vaccinations, I just shake my head. I can’t believe it…. We’re all in it together and we should realize that…. That’s the way a civilized society should function. We should take care of each other.” (Interview 16, patient and caregiver, ADI of 2)

## Discussion

This qualitative study, incorporating both patient and caregiver perspectives, offers important insights on how SDOH both negatively and positively impact recovery from acute illnesses, such as COVID-19. Socioeconomic and racial disparities are the major underlying social condition of the syndemic with COVID-19 in the US, and SDOH are an important mechanism of these disparities. We identified several resources and challenges across SDOH domains, with no major differences by participant ADI, and 3 emergent cross-cutting themes that help illuminate how SDOHs influence recovery: mobilization and innovation of resources, navigating destabilization and change introduced by illness, and mistrust of established institutions. These findings build on existing literature regarding the impact of SDOH on illness recovery, which has mainly focused on mental illness, critical illness, and/or quantitative data.^33,34,35,36,37,38^ The cross-cutting themes represent factors that modify an individual’s interaction with social resources and that may be valuable additions to existing conceptual models of SDOH (Box). For example, a patient may have access to health care, but whether and how the patient uses that access is modified by trust.^39,40,41^ Health systems are making unprecedented investments in programs to identify and address health-related social needs, but the ultimate impact of these programs is highly dependent on patients’ perceptions of the trustworthiness of health systems.^42^ Thus, simultaneous efforts to demonstrate and foster trustworthiness, such as partnering with and respecting the expertise of community-based organizations and diversifying health system workforces to better represent the populations they serve, will be critical.^43,44,45^ Similarly, how patients access social resources during times of illness may depend on whether those resources can be mobilized in innovative ways to meet patients’ new and evolving needs. There may be important lessons to learn from the unprecedented pace of innovation that was seen during the COVID-19 pandemic, such as in telehealth reimbursement policies.^46,47,48^ Creating health care structures that can continue to support rapid-cycle innovation will be important in optimally integrating social and medical care.

Box. **Adapted Framework of Social Determinants of Health**

**Social Factors**

Educational access and quality
Health care access and quality
Neighborhood and built environment
Social and community context
Economic stability

**Cross-Cutting Themes**

Mobilization and innovation of resources
Navigating changes brought on by illness
Trust and mistrust of established institutions

Our findings also speak to the dynamism of SDOH in the context of acute illness and recovery and underscore the importance of qualitative data in understanding the differential effects of these factors. SDOH are often quantified using static measures; however, it is known that SDOH change, especially during and following acute health events.^9^ For example, an individual’s neighborhood and home resources may change if they must move in with a family member following illness. Static measures also obscure how the same social factors may be associated with a positive outcome for one individual and a negative outcome for another individual. For example, some participants in our study described how stairs served as a barrier to accessing their home, while others described how stairs separating floors of their home allowed them to quarantine and prevent others in their home from getting ill.

Notably, regardless of ADI, most participants in our study described accessing positive social resources. This underscores the need to take a strengths-based approach to measuring SDOH, in which health systems and health care professionals identify resources available to their patients and community that could be leveraged to support illness recovery. One example of this is NCCARE360,^49^ an electronic referral coordination platform that helps health care professionals and organizations connect individuals and families to community resources that can support their physical and mental health. Formed in January 2019, it has centralized case management for over 8000 individuals and families.

Finally, this study found shortcomings of existing measures used for conceptualizing evaluation of SDOH. For example, quantitative measures, such as ADI, may not fully capture the range of social needs that individuals experience and may be poor surrogates for individual experiences. They also may miss important intersections of various biological and social factors associated with health outcomes. We found that individual experiences of SDOH did not differ by ADI, suggesting that ADI may not be a helpful tool for assessing SDOH impact on illness recovery. Similarly, Bensken et al^50^ found that area-level SDOH measures, including ADI, the Social Deprivation Index, and the Material Community Deprivation Index, had low accuracy and sensitivity in predicting individual social needs. While these area-level assessments of SDOH can be helpful in standardizing the way that social risks are measured, health systems and policymakers will need to plan for further follow-up to identify specific individual-level risks as well as strategies to help address those risks. Furthermore, the ability to identify and respond to larger societal phenomena, such as syndemics, will likely require qualitative data.

### Strengths and Limitations

Strengths of this study include that we were able to obtain first-person perspectives from both patients and caregivers, which allowed for more in-depth discussion of SDOH domains and deeper insights into the impact of SDOH on COVID-19 recovery. There are also some limitations. This was a single-site study in a distinct geographic area with a largely college-educated adult population that was able to access a specialized pulmonary clinic, potentially limiting the generalizability of our findings, especially to children or mixed-age populations. Our findings may not reflect the experiences of individuals who may not have been able to access and establish care in a specialized clinic at a tertiary hospital or among individuals with conditions that have a different recovery trajectory than an acute respiratory illness. We also interviewed a greater proportion of participants from low-disadvantage neighborhoods (ie, high socioeconomic status), limiting our ability to quantify significant differences in SDOH constructs by ADI. The use of videoconferencing software and telephone calls to conduct interviews may also have affected individuals’ willingness to participate as well as the rapport, trust, and information sharing. In addition, the time-varying nature of COVID-19 and our single-time interview limited our ability to assess how participants’ thoughts and needs may have changed or evolved longitudinally throughout their recovery.

## Conclusions

In this qualitative study of individuals recovering from COVID-19 and their caregivers, participants identified distinct SDOH that impacted recovery from acute illness. The findings suggest that the same SDOH domain may support 1 individual while hindering recovery for another. Themes that cross-cut SDOH domains may help to explain mechanisms of the COVID-19 syndemic and experiences of recovery. Results from our study support the need to collect both quantitative and qualitative longitudinal, multidomain data on SDOH for patients. These data can be used to identify patients at greatest risk for adverse outcomes as well as determine opportunities for system- and policy-level interventions that can mitigate the outcomes of long-standing structural inequities that impact health.

## Notes

Supplement 1. **eAppendix 1.** Interview Guide **eAppendix 2.** Final Codebook **eTable.** Frequency of Discussion of Social Determinants of Health by Area Deprivation Index of Patient’s Home Address

Supplement 2.
Data Sharing Statement
