# Supplementary material for: Role of Social Determinants of Health in COVID-19 Recovery: A Qualitative Study
Source: JAMA Netw Open. 2025 Jan 6;8(1):e2453261. doi: 10.1001/jamanetworkopen.2024.53261 (PMC11704979; doi:10.1001/jamanetworkopen.2024.53261)
Supplement: Supplement 1. — eAppendix 1. Interview Guide eAppendix 2. Final Codebook eTable. Frequency of Discussion of Social Determinants of Health by Area Deprivation Index of Patient’s Home Address [file jamanetwopen-e2453261-s001.pdf]

Supplementary Online Content

Navuluri N, Bhavsar NA, Chen V, et al. Role of social determinants of health in COVID-19 recovery: a qualitative study. *JAMA Netw Open*. 2025;8(1):e2453261. doi:10.1001/jamanetworkopen.2024.53261

eAppendix 1. Interview Guide

eAppendix 2. Final Codebook

eTable. Frequency of Discussion of Social Determinants of Health by Area Deprivation Index of Patient's Home Address

This supplementary material has been provided by the authors to give readers additional information about their work.

© 2025 Navuluri N et al. *JAMA Network Open*.

eAppendix 1: Interview guide

1. Tell me about your health before COVID. What did you do in a typical day?
2. During your recovery from COVID, what COVID-related symptoms were most bothersome to you? By bothersome I mean those that limited you from doing what you wanted to do at the time.
    a. What symptoms - brain fog, headaches, fatigue, loss of senses, fevers, myalgia (body aches), respiratory, other
    b. How did these symptoms impact your day to day?
    c. How long did it take you to feel 'back to normal'?
        i. PROBE: what were some of the things in your environment or community that helped you get 'back to normal'? Or back to your 'usual' roles in your family/community?
    d. *To the caregiver:* Is there anything else you would add about (NAME)'s symptoms and/or the impact of those symptoms?
3. Tell me about your home.
4. Thinking about your home, what were some things that made it harder for you to recover from COVID? How did you work through these challenges?
    a. *To the caregiver: What else would you add?*
5. Thinking about your home, what were some things that helped you when you were recovering from COVID?
    a. *To the caregiver: What else would you add?*
6. Tell me about the area where you live.
    a. PROBES (for all questions in this section): urban/rural, safe/unsafe, appearance, green space, density, transportation, proximity to services/retail stores, access to medical care
7. Thinking about the area where you live, what were some things that made it harder for you to recover from COVID? How did you work through these challenges?
    a. *To the caregiver: What else would you add?*
8. Thinking about the area where you live, what were some resources that helped you when you were recovering from COVID?
    a. *To the caregiver: What else would you add?*
9. Tell me about where you work.
    a. PROBES (for all questions in this section): paid time off, remote work, flexible hours, gym, healthy food options
10. Thinking about the place where you work, what were some things that made it harder for you to recover from COVID? How did you work through these challenges?
    a. *To the caregiver: What else would you add?*
11. Thinking about the place where you work, what were some resources that helped you when you were recovering from COVID?
    a. *To the caregiver: What else would you add?*

We've talked a lot about physical spaces that were part of your COVID recovery. Now let's focus on the social and emotional aspects of recovery.

12. How did your community, including your family, friends, and church family, support you while you were recovering from COVID?
    a. PROBE: informational, tangible support
    b. *To the caregiver: What else would you add?*

© 2025 Navuluri N et al. *JAMA Network Open*.

13. Please take a look at this picture that describes the things we each need to be healthy and recover from illness. Based on your experience, what are some specific things you think might be helpful to patients during recovery from COVID?
    a. Access to health care
    b. Food
    c. Access to education
    d. Neighborhood and build environment
    e. Financial stability
    f. Social and community support
14. What else is important for us to know about what it’s like to recover from COVID in your neighborhood?

© 2025 Navuluri N et al. *JAMA Network Open*.

eAppendix 2: Final codebook

Code	Description	Likely SDOH Domains
Having pre-existing condition	Patient and/or caregiver discusses preexisting condition that impacted COVID and COVID recovery.	Access to healthcare
Experiencing hospitalization	Patient and/or caregiver talks about the patients experience while hospitalized for COVID.	Access to healthcare
Experiencing COVID symptoms	Patient and/or caregiver discusses specific COVID symptoms that the patient found problematic during recovery	Access to healthcare
Describing home	Patient and/or caregiver description of the physical, emotional, and social (including the people in the home) aspects of the place they call home and recovered from COVID. This includes proximity to retail and services, safety, access to medical care, transportation, etc.	Neighborhood and build environment
Living in neighborhood	Patient and/or caregiver talk about issues related to neighborhood and the built environment that will help patients during recovery from COVID. These issues may be related to their own personal experience or their general thoughts about other COVID patients	Neighborhood and build environment
Facilitating recovery	Patient and/or caregiver discusses factors in the home, neighborhood, town, city surrounding their home that helped them during their recovery from COVID. This can include services, distances, physical environment, and specific resources.	All
Experiencing challenges	Patient and/or caregiver discusses factors in the home, neighborhood, town, city surrounding their home that made recovery more difficult. This can include people, services, distances, physical environment, etc.	All
Addressing challenges	Patient and/or caregiver talk about the solutions they developed or resources they used to address the challenges they experienced during recovery from COVID.	All
Dealing with work	Patient and/or caregiver talk about job related factors that helped and were challenging when recovering from COVID.	Financial stability; Education
Addressing challenges with work	Patient and/or caregiver talk about solutions they developed or resources they used to address job related challenges while recovering from COVID	Financial stability; education
Getting support	Patient and/or caregiver talk about emotional, informational, tangible support received from family, friends, church, community.	Social and community support
Having access to care	Patient and/or caregiver talk about issues related to access to care that will help patients during recovery from COVID. These issues may be related to their own	Access to healthcare

© 2025 Navuluri N et al. *JAMA Network Open*.

	personal experience or their general thoughts about other COVID patients	
Accessing food	Patient and/or caregiver talk about issues related to access to food that will help patients during recovery from COVID. These issues may be related to their own personal experience or their general thoughts about other COVID patients	Food
Accessing education	Patient and/or caregiver talk about issues related to access to education and training that will help patients during recovery from COVID. These issues may be related to their own personal experience or their general thoughts about other COVID patients	Education
Having financial stability	Patient and/or caregiver talk about issues related financial stability that will help (or hurt) patients during recovery from COVID	Financial stability
Having social support	Patient and/or caregiver talk about issues related to social support that will help (or hurt) patients during recovery from COVID. This is more for looking forward about what might help and/or when talking about general population.	Social and community support
Interesting	Use this code to mark any segment of text that stand out to you as interesting.	All

© 2025 Navuluri N et al. *JAMA Network Open*.

eTable: Frequency of discussion of social determinants of health (SDOHs) by area deprivation index (ADI) of patient's home address

**How often SDOH domains were discussed in interviews, n (column %)**			
	High ADI (high disadvantage)<br>N = 10 interviews	Low ADI (low disadvantage)<br>N = 14 interviews	Total<br>N = 24 interviews
Neighborhood and built environment	83 (26.2)	135 (36.1)	218 (31.5)
Social and community context	75 (23.7)	86 (23.0)	161 (23.3)
Economic stability	63 (19.9)	70 (18.7)	133 (19.2)
Health care	48 (15.1)	41 (11.0)	89 (12.9)
Food access and quality	29 (9.1)	23 (6.1)	52 (7.5)
Education access and quality	19 (6.0)	19 (5.1)	38 (5.5)

**How SDOH domains were discussed in interviews, n (column %)**			
	High disadvantage ADI<br>N = 10 interviews	Low disadvantage ADI<br>N = 14 interviews	Total<br>N = 24 interviews
Neighborhood and built environment			
Positive	7 (70.0)	9 (64.3)	16
Negative or ambivalent	2 (20.0)	4 (28.6)	6
Not discussed	1 (10.0)	1 (7.1)	2
Social and community context			
Positive	4 (40.0)	5 (35.7)	9
Negative or ambivalent	6 (60.0)	9 (64.3)	15
Not discussed	0	0	0
Economic stability			
Positive	9 (90.0)	10 (71.4)	19
Negative or ambivalent	1 (10.0)	3 (21.4)	4
Not discussed	0	1 (7.1)	1
Health care			
Positive	5 (50.0)	7 (50.0)	12
Negative or ambivalent	4 (40.0)	7 (50.0)	11
Not discussed	1 (10.0)	0	1
Food access and quality			
Positive	5 (50.0)	9 (64.3)	14
Negative or ambivalent	5 (50.0)	4 (28.6)	9
Not discussed	0	1 (7.1)	1
Education access and quality			
Positive	2 (20.0)	2 (14.3)	4
Negative or ambivalent	3 (30.0)	1 (7.1)	4
Not discussed	5 (50.0)	11 (78.6)	16

© 2025 Navuluri N et al. *JAMA Network Open*.
