# Supplementary material for: Role of Social Determinants of Health in COVID-19 Recovery: A Qualitative Study
Source: JAMA Netw Open. 2025 Jan 6;8(1):e2453261. doi: 10.1001/jamanetworkopen.2024.53261 (PMC11704979; doi:10.1001/jamanetworkopen.2024.53261)
Supplement: Supplement 2. — Data Sharing Statement [file jamanetwopen-e2453261-s002.pdf]

Data Sharing Statement

Navuluri. Role of Social Determinants of Health in COVID-19 Recovery. *JAMA Netw Open*. Published January 06, 2025. doi:10.1001/jamanetworkopen.2024.53261

Data
Data available: Yes
Data types: Deidentified participant data
How to access data: neelima.navuluri@duke.edu
When available: With publication

Supporting Documents
Document types: None

Additional Information
Who can access the data: researchers whose proposed use of the data has been approved
Types of analyses: For qualitative analyses
Mechanisms of data availability: With a signed data access agreement
